# Photothermal and Photodynamic Strategies for Diagnosis and Therapy of Alzheimer’s Disease by Modulating Amyloid-β Aggregation

**DOI:** 10.3390/bios15080480

**Published:** 2025-07-24

**Authors:** Fengli Gao, Yupeng Hou, Yaru Wang, Linyuan Liu, Xinyao Yi, Ning Xia

**Affiliations:** 1 Henan Province Key Laboratory of New Opto-Electronic Functional Materials, College of Chemistry and Chemical Engineering, Anyang Normal University, Anyang 455000, China; flgao@aynu.edu.cn (F.G.); 16692208021@163.com (Y.H.); w13849889971@163.com (Y.W.); 2 College of Chemistry and Chemical Engineering, Central South University, Changsha 410083, China; 222311028@csu.edu.cn (L.L.); yixinyao@csu.edu.cn (X.Y.)

**Keywords:** Alzheimer’s disease, amyloid-β, phototherapy, photothermal therapy, photodynamic therapy, aggregation-induced emission

## Abstract

Amyloid-β (Aβ) aggregates are considered as the important factors of Alzheimer’s disease (AD). Multifunctional materials have shown significant effects in the diagnosis and treatment of AD by modulating the aggregation of Aβ and production of reactive oxygen species (ROS). Compared to traditional surgical treatment and radiotherapy, phototherapy has the advantages, including short response time, significant efficacy, and minimal side effects in disease diagnosis and treatment. Recent studies have shown that local thermal energy or singlet oxygen generated by irradiating certain organic molecules or nanomaterials with specific laser wavelengths can effectively degrade Aβ aggregates and depress the generation of ROS, promoting progress in AD diagnosis and therapy. Herein, we outline the development of photothermal therapy (PTT) and photodynamic therapy (PDT) strategies for the diagnosis and therapy of AD by modulating Aβ aggregation. The materials mainly include organic photothermal agents or photosensitizers, polymer materials, metal nanoparticles, quantum dots, carbon-based nanomaterials, etc. In addition, compared to traditional fluorescent dyes, aggregation-induced emission (AIE) molecules have the advantages of good stability, low background signals, and strong resistance to photobleaching for bioimaging. Some AIE-based materials exhibit excellent photothermal and photodynamic effects, showing broad application prospects in the diagnosis and therapy of AD. We further summarize the advances in the detection of Aβ aggregates and phototherapy of AD using AIE-based materials.

## 1. Introduction

Alzheimer’s disease (AD) is the most common cause of dementia, characterized by the gradual decline and irreversible damage of patients’ memory, communication skills, and cognitive abilities. As of now, at least 47 million people worldwide are suffering from this disease [1], but the exact pathology of AD remains unclear. The currently used drugs can relieve the symptoms and slow down the progression of AD, but they cannot reverse the pathology of AD [2]. Since amyloid-β (Aβ) peptides, mainly including Aβ40 (~90%) and Aβ42 (~9%), were isolated from the brains of AD patients, the “amyloid cascade hypothesis” has become a research hotspot [3,4,5]. This hypothesis suggests that the production, accumulation, and deposition of Aβ are the main causes of AD [6,7]. Aβ is one of the normal products of cellular metabolism, while its aggregation is a pathological process [8]. Due to the failed clearance or excessive abnormal production, Aβ peptides accumulate in the brain and subsequently self-assemble into toxic aggregates, including oligomers, protofibrils, and mature fibrils, ultimately resulting in neuronal death [9,10,11]. The self-assembly process is mainly based on noncovalent interactions, such as van der Waals forces, hydrogen bonding, electrostatic interactions, and hydrophobic interactions. With the gradual deepening of AD research, inhibiting Aβ aggregation or eliminating Aβ aggregates has become a feasible treatment method [12]. The current anti-amyloidosis methods, including photooxidation, copper chelators, aggregation inhibitors, and thermal dissociation, have become treatment options for AD [13]. For example, different inhibitors have been reported to inhibit Aβ aggregation and/or dissociate Aβ aggregates, including peptides or peptide mimetics, small organic molecules, nanomaterials, and Aβ-specific antibodies. However, most of them have not yet been clinically applied for the effective treatment of AD. New strategies such as nerve regeneration, gene editing, gene silencing, immunotherapy, implantable nerve devices, and non-pharmacological interventions have also been developed. Most of these methods have shown promising results under controlled laboratory conditions, but they have failed in clinical trials [14,15]. Thus, the design of new therapeutic strategies and drugs for AD diagnosis and treatment is still necessary.

Phototherapy, an innovative therapeutic method relying on the utilization of light to produce heat or reactive oxygen species (ROS) by photoresponsive agents, has been applied to the treatment of various diseases in clinical dermatology, ophthalmology, and oncology [16]. Its advantages, including operational flexibility, low side effects, non-invasiveness, and high spatiotemporal resolution have shown enormous potential in the treatment of AD [17]. Phototherapy mainly consists of two types: photothermal therapy (PTT) and photodynamic therapy (PDT). In the former, the interaction between photothermal transduction agents and light can generate a thermal effect, creating high local heat to disrupt physiological conditions promoting Aβ aggregation or dissolve Aβ deposits, thereby achieving the effect of PTT [18]. Moreover, the produced local heat can upregulate the expression of heat shock proteins to regulate protein folding and lower ROS concentrations. Therefore, PTT has shown great potential in AD therapy. In PDT, photosensitizers are excited by light and interact with surrounding oxygen or other molecules to generate ROS. These oxidative species can react with amino acid residues and prevent Aβ self-assembly, therefore regulating Aβ aggregation. Due to its outstanding advantages of minimal invasiveness, limited side effects, and spatiotemporal control, PDT has become a promising strategy to enhance the therapeutic efficiency of AD. According to the differences in the excitation lights of photosensitizers, the method of PDT can be divided into three categories: ultraviolet light, visible light, and near-infrared light (NIR) [18]. Generally, lasers with stronger intensity and longer wavelengths tend to penetrate deeper. For example, compared to the more commonly used NIR-I laser, NIR-II light has biological advantages such as deeper penetration and greater maximum allowable exposure [19,20]. In addition, it has been reported that the combination of phototherapy and fluorescence imaging technology can achieve highly effective therapeutic results. However, traditional fluorophores may suffer from the interference of the aggregation-caused quenching (ACQ) effect, severely limiting their bioimaging applications. Since the concept of aggregation-induced emission (AIE) was first coined by Tang and co-workers, AIE luminogens (AIEgens) have been widely used as fluorescence probes in fluorescence imaging-guided phototherapy [21]. More importantly, through rational optimization of molecular structure, AIEgens can simultaneously produce fluorescence and heat or ROS under excitation, promoting the development of imaging-guided synergistic therapies. Furthermore, although many efforts have been made to synthesize molecular drugs for phototherapy, the blood-brain barrier (BBB) greatly limits their application in the diagnosis and treatment of AD [22,23]. The design of imaging agents and therapeutic drugs that can cross the BBB still remains a major challenge [24,25]. Nanomaterials have been widely used to load photoreponsive agents and increase the therapeutic efficiency due to the enhanced permeability and retention effect. In particular, some nanomaterials showing photoresponsive properties can inhibit Aβ aggregation and/or disassemble Aβ aggregates, which can facilitate the development of multifunctional therapeutic strategies for AD.

Currently, there are several excellent reviews on the application of photoresponsive materials in the diagnosis and treatment of diseases, including AD [26,27,28,29,30,31,32]. For example, Lee et al. introduced the achievements and working principles of light-triggered modulation of Aβ self-assembly based on photosensitive materials and platforms [28]. Xu et al. summarized the PDT methods for AD treatment from molecular catalysis to photo-nanomedicine [30]. However, these reviews that mainly focus on PDT do not involve PTT strategies. Although Yang’s group reviewed the progress of phototherapy methods in AD treatment using NIR-activated reagents [31,32], their work do not cover the reagents activated by ultraviolet and visible light. Liu et al. outlined photoresponsive materials that can regulate Aβ aggregation in 2021 [29], but in the past five years, more new materials and strategies have been designed and reported. Therefore, it is necessary to systematically outline the development of PTT and PDT strategies for regulating Aβ aggregation as well as fluorescence imaging-guided phototherapy of AD. In this review, we describe different methods for treating AD by inhibiting Aβ aggregation and decomposing Aβ fibers from the perspectives of PDT and PTT. Considering the remarkable progress of imaging technology, we also summarized the progress of AIE-based fluorescence imaging-guided PTT and PDT therapies. Finally, we summarized the current challenges and prospects in promoting light-activated regulation of Aβ aggregation for AD treatment.

## 2. PTT Methods

Photothermal agents can generate hyperthermia by absorbing light energy and converting it into thermal energy [33]. PTT can lead to local temperature increase on the treatment sites, typically utilizing NIR light to activate photothermal agents [20,26,27,28,29,30,34,35,36]. It can offer a greater degree of spatiotemporal control in contrast to the systemic therapy, thereby reducing off-target toxicity. At the cellular level, this process can affect the activity of certain proteins [37]. Mild hyperthermia can temporarily increase vascular permeability at the vascular level, enhancing the transfer and accumulation of photothermal agents at the treatment sites [38]. The formation of amyloid proteins is highly dependent on many factors, such as pH, temperature, concentration, and ionic strength [39]. NIR-responsive photothermal agents for AD treatment represent an effective approach to enhance BBB permeability and dissolve Aβ deposits in the brain [31,32,40]. Consequently, various photothermal materials have been developed to regulate the aggregation of Aβ, such as polymers, organic self-assemblies, carbon-based materials, metal-based nanomaterials, and two-dimensional materials [41,42,43,44].

### 2.1. Polymeric and Self-Assembled Nanoparticles

**Figure 1:**
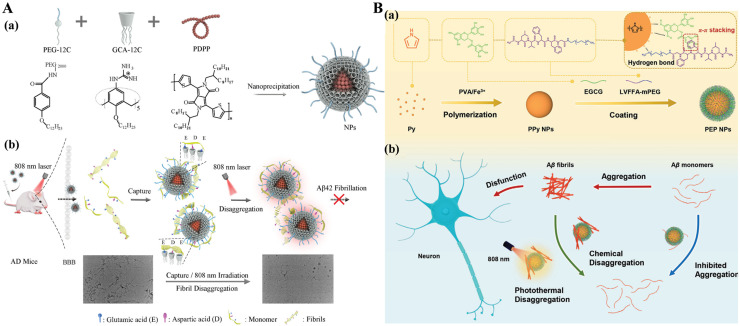
(**A**) (**a**) Assembly route of PEP NPs. (**b**) Schematic representation of the inhibition and disaggregation ability of NPs on Aβ_42_ fibrillation and elimination of Aβ_42_ plaques in AD mouse brain. Reprinted with permission from ref. [47]. Copyright 2021 Wiley-VCH. (**B**). Schematic representation of (**a**) the synthesis of PEP NPs and (**b**) the NIR light-induced synergistic inhibition and disaggregation of Aβ fibrils. Reprinted with permission from ref. [48]. Copyright 2024 Wiley-VCH.

Polymer nanoparticles are considered superior to other materials in medical applications due to their good biodegradability and biocompatibility. The biological function, molecular weight, and structural architecture of polymer nanoparticles can be easily manipulated to meet the requirements of Aβ inhibitors [45,46]. Some polypyrrole polymers show strong absorption in the NIR region and exhibit high photothermal conversion efficiency as well as deep tissue penetrability. They have been utilized as photothermal materials to prevent Aβ fibrillation and improve the crossing capability of nanoparticles passing through the BBB. For example, Geng et al. prepared photothermal materials by one-pot co-precipitation of 4-(dodecyloxy)benzamido-terminated methoxy poly(ethylene glycol) (PEG), amphiphilic guanidinocalix[5]arene (GC5A), and poly-5,5′-(2,5-bis(2-octyldodecyl)3,6-di(thiophen-2-yl)-2,5-dihydropyrrolo[3,4-c] pyrrole-1,4-dione (PDPP) (Figure 1A) [47]. The NIR light-responsive nanoparticles can selectively bind to Aβ, inhibit Aβ fibrillation, and disaggregate Aβ fibrils. In this method, GC5A showed the ability to bind the Glu and Asp residues in Aβ via the host-guest interaction. PEG was used to enhance the stability and biocompatibility of the nanoparticles. PDPP as an NIR-responsive reagent promoted the disaggregation of Aβ fibrils by the generation of local heat. The polymeric nanoparticles showing good BBB permeability can reduce the cytotoxicity of Aβ fibrils and ultimately eliminate Aβ plaques in the brains of AD mice after NIR irradiation. In this work, immunofluorescence imaging was used to investigate the Aβ plaque-clearing ability of NPs on the brains of 3-month-old 5xFAD mice. In addition, Zhang et al. prepared NIR-responsive photothermal nanoparticles named PEP NPs through the polymerization of pyrrole monomers, the co-assembly of Aβ-binding LVFFA peptide linked with methoxypolyethylene glycol amine (mPEG), and the coating of aggregation inhibitor epigallocatechin gallate (EGCG) (Figure 1B) [48]. The LVFFA peptide on the nanoparticle surface can selectively target Aβ, and the coated EGCG can inhibit Aβ fibrillization. The disaggregation efficiency of PEP NPs for Aβ fibrils reached 38%. After irradiation by NIR light, more Aβ fibrils were disaggregated in a shorter time period. In addition, the photothermal PEP NPs significantly decreased the toxicity of Aβ fibrils to PC12 cells by preventing the Aβ-induced disruption of cell membranes.

Hydrophobic phenylalanine residues play a vital role in the self-assembly of peptides, including Aβ, facilitating the preparation of self-assembled materials with various shapes. Porphyrin derivatives are a type of photosensitizer approved by the FDA for photodynamic therapy of tumors. Our group found that 5-(4-carboxyphenyl)-10,15,20-triphenylporphyrin (TPP)-substituted phenylalanine-phenylalanine dipeptide (TPP-FF) could self-assemble into spherical nanoparticles. The TPP-FF nanoparticles can prevent Aβ aggregation, disassemble Aβ fibrils under light illumination, and depress the generation of ROS. Moreover, the nanoparticles can be used as the nanocarriers and quenchers of dye-labeled aptamers for the selective detection of Aβ oligomers.

### 2.2. Carbon-Based Materials

**Figure 2:**
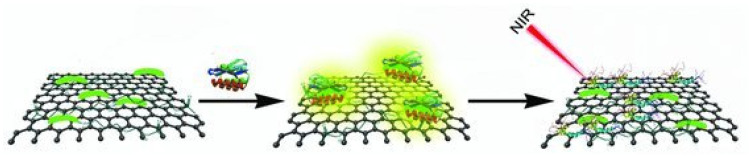
Schematic illustration of ThS-modified GO with high NIR absorbance. Reprinted with permission from ref. [52]. Copyright 2012 Wiley-VCH.

Carbon-based materials are readily available substances with high potential in various fields, including graphite, graphene, carbon nanotubes, fullerenes, and other carbon forms (carbon nitride, carbon aerogels, etc.) [49,50,51]. They have been widely used in phototherapy due to their low cost, ease of preparation, good chemical inertness, effective light absorption in the NIR region, and high light conversion efficiency. Graphene is a two-dimensional crystalline carbon with unique electronic and optical properties. The high absorption of graphene in the NIR region has facilitated its biomedical applications as a photothermal material for cancer therapy. Qu’s group for the first time investigated the application prospects of graphene-based materials for photothermal treatment of AD (Figure 2) [52]. In their work, graphene oxide (GO) was modified with thioflavin-S (ThS) to produce local heat and disaggregate Aβ aggregates under low-power NIR laser irradiation. ThS was conjugated with GO to selectively bind Aβ aggregates. The GO–ThS conjugates can efficiently reduce Aβ-induced cytotoxicity toward PC12 cells upon NIR irradiation.

**Figure 3:**
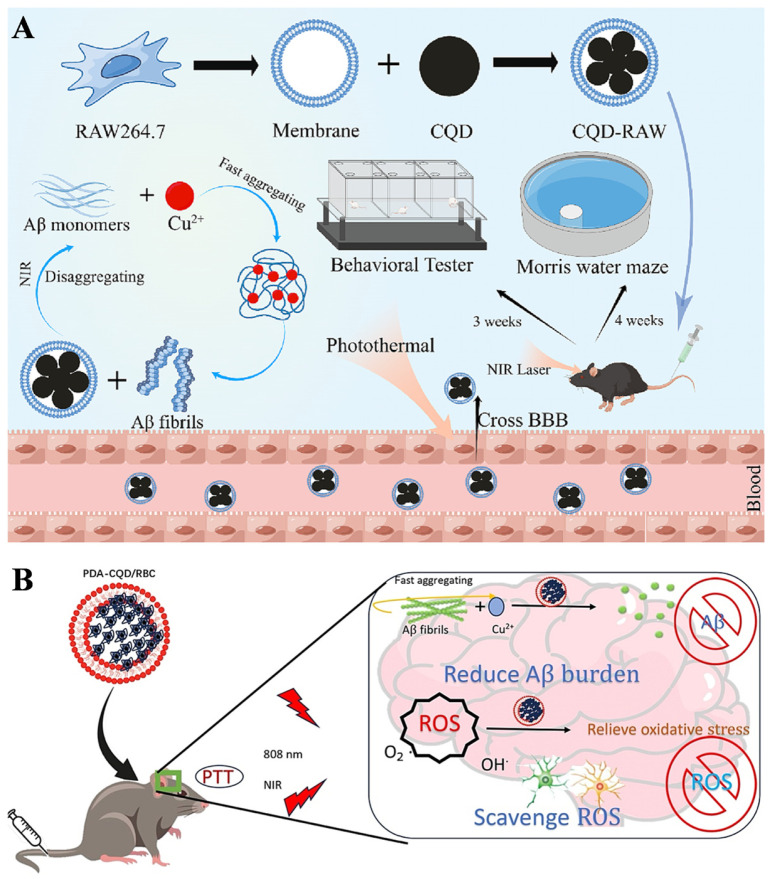
(**A**) Schematic illustration of CQD-RAW preparation and CQD-RAW nanosystem for AD treatment. Reprinted with permission from ref. [53]. Copyright 2023 Elsevier B.V. (**B**) Schematic illustration of PDA-CQD/RBC nanosystem for AD combination therapy. Reprinted with permission from ref. [54]. Copyright 2024 Elsevier B.V.

Carbon quantum dots (CQDs) are a remarkable class of carbon-based nanomaterials that have great potential for interdisciplinary applications in medical diagnosis and drug delivery. Recent studies suggested that CQD-based hybrid materials can effectively prevent the fibrillization of Aβ, promoting the diagnosis and treatment of AD. For example, Ye et al. proposed a strategy for Aβ-targeted treatment of AD by using macrophage membrane (RAW-M) to encapsulate nitrogen-doped CQD (Figure 3A) [53]. The nitrogen-containing moieties on the CQD surface favored the capture of free Cu^2+^ and the inhibition of Aβ aggregation. The formed Aβ fibrils could be disassembled by CQD due to its excellent photothermal property under NIR irradiation. The in vitro and in vivo brain fluorescence imaging experiments indicated that CQD-RAW exhibited excellent BBB permeability and could strongly reduce Aβ deposition, mitigate neuroinflammation, and improve memory and learning ability, providing an effective strategy for Aβ-targeted treatment of AD. In addition, local heating caused by NIR laser irradiation can decompose the formed Aβ fibers and enhance the BBB permeability, which has been confirmed by whole-body fluorescence imaging. Liu et al. prepared a NIR nanocomposite for AD treatment by using red blood cell membrane (RBC) to encapsulate CQD and polydopamine (PDA) (Figure 3B) [54]. The resulting PDA-CQD/RBC nanocomposite could dismantle Aβ fibrils and enhance BBB permeability by local heating from NIR irradiation. In this work, RBC showed anti-immunorecognition properties toward immune clearance, PDA exhibited enzyme-like activity to alleviate oxidative stress damage, and CQD as chelating agents could sequester Cu^2+^ to prevent metal ion-promoted Aβ aggregation. The experiment results in vitro and in animals further demonstrated that the PDA-CQD/RBC nanocomposite exhibited neuroprotective functions by improving the behavioral response of AD mice and enhancing memory, cognitive, and learning abilities.

**Figure 4:**
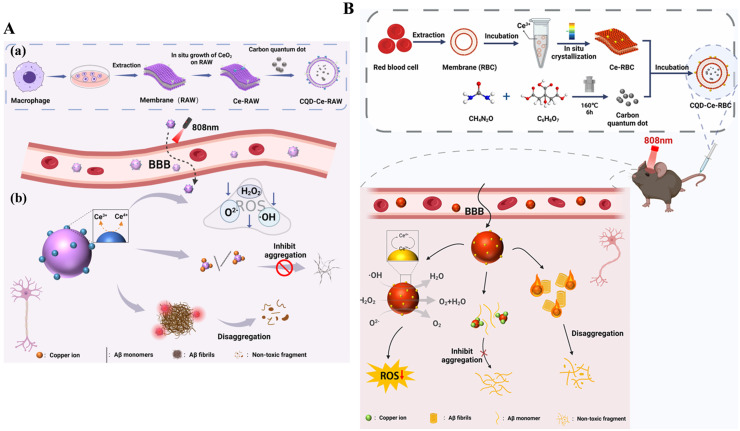
(**A**) (**a**) Preparation pathway of CQD-Ce-RAW. (**b**) Schematic illustration of the multi-pathway therapeutic mechanism of CQD-Ce-RAW in combination with PTT for AD. Reprinted with permission from ref. [55]. Copyright 2023 Elsevier B.V. (**B**) Schematic illustration of CQD-Ce-RBC synthesis and synergistic PTT for multi-targeting against AD. Reprinted with permission from ref. [56]. Copyright 2020 American Chemical Society.

In addition, Chi et al. reported a multifunctional nanocomposite prepared by in-situ growth of CeO_2_ nanoparticles on macrophage membrane (Ce-RAW) for the modification of CQD (Figure 4A) [55]. The resulting CQD-Ce-RAW showed excellent photothermal properties and therapeutic efficacy. The macrophage membrane endowed the nanocomposite with good anti-phagocytic and anti-inflammatory functions. CeO_2_ nanoparticles on the membrane as ROS scavengers efficiently alleviated the oxidative stress. CQD, serving as a photosensitizer efficiently promoted the dissociation of Aβ aggregates, chelated free Cu^2+^, and inhibited Aβ aggregation, which was also confirmed by cell and animal experiments using 1,1′-dioctadecyl-3,3,3′,3′-tetramethylindodicarbo cyanine perchlorate (DID)-labeled CQD-Ce-RAW for fluorescence imaging of mouse brain tissues. At the same time, Chi et al. prepared CQD-Ce-RBC nanocomposites for AD treatment using red blood cell membranes (RBC) as templates for the in-situ growth of CeO_2_ nanocrystals and encapsulation of CQD (Figure 4B) [56]. RBC enhanced the biocompatibility and improved the immune evasion of nanocomposites. CQD prevented Cu^2+^-promoting Aβ aggregation, promoted the disassembly of Aβ fibrils, and improved the BBB permeability with its thermal effect, which was confirmed by imaging the dissected brains using the In Vivo Xtreme II imaging system.

**Figure 5:**
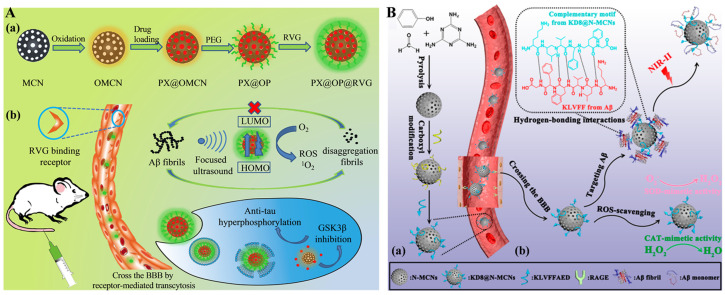
(**A**) Schematic illustration of the PX@OP@RVG nanosystem (**a**), and NPs crossing the BBB by receptor-mediated transcytosis and double-targeted treatment process in vitro and in vivo (**b**). Reprinted with permission from ref. [58]. Copyright 2018 American Chemical Society. (**B**) Schematic illustration of KD8@N-MCNs synthesis (**a**) and mechanism action of KD8@N-MCNs (**b**). Reprinted with permission from ref. [59]. Copyright 2020 American Chemical Society.

Owing to their unique structure and outstanding photothermal conversion efficiency, mesoporous carbon nanoparticles and related derivatives have been widely explored in disease treatment. The customized mesoporous carbon nanoparticles with adjustable channels and intelligent surface modification can be used to deliver various kinds of active agents and improve the limitations of monotherapy, including severe side effects and poor therapeutic efficacy [57]. Light in the second NIR region (NIR-II) has deeper tissue penetration, a lower signal-to-noise ratio, and a higher maximum allowable exposure than the commonly used excitation light. Therapeutic efficacy can be improved by changing the short wavelength of excitation light to NIR-II light. Xu et al. reported a multifunctional nanodrug with multiple targets to treat AD based on protoporphyrin IX (PX)-modified oxidized mesoporous carbon nanospheres (OMCN) (Figure 5A) [58]. The resulting PX@OMCN was modified with aminated poly(ethylene glycol) (PEG) via a covalent coupling reaction to obtain PX@OP. The modification of RVG peptide and the photothermal effect of nanodrugs under 750 nm NIR irradiation significantly improved the safety and efficiency of PX across the BBB. The nanodrugs can inhibit tau phosphorylation, and PX can trigger the production of ROS and inhibit Aβ aggregation under ultrasound, which was substantiated by fluorescence distribution and thermal imaging of the brains of AD mice. The cognitive levels of AD mice were improved due to the dual-target inhibition. In addition, Ma et al. reported a method for phototherapy of AD using KLVFFAED peptide-modified N-doped three-dimensional mesoporous carbon nanospheres (KD8@N-MCNs) as the NIR-II PTT agents (Figure 5B) [59]. The Aβ aggregates were photothermally decomposed through dense scalp and skull, and the aggregation of Aβ was inhibited by binding to KD8@N-MCNs through the hydrogen bonding, hydrophobic, van der Waals, and electrostatic interactions. More interestingly, KD8@N-MCNs could deplete intracellular ROS and neuroinflammation based on their superoxide dismutase and catalase-like activities. The PTT materials exhibited excellent BBB permeability through Aβ transportation-mimicked pathway, which was monitored by using cyanine5 amine-modified KD8@N-MCNs (named Cy5/KD8@N-MCNs) as the probes. The in vivo investigation suggested that KD8@NMCNs can alleviate Aβ burden in the brains of model mice and rescue their memory function and learning ability.

### 2.3. Metal-Based Materials

#### 2.3.1. Metal Nanomaterials

**Figure 6:**
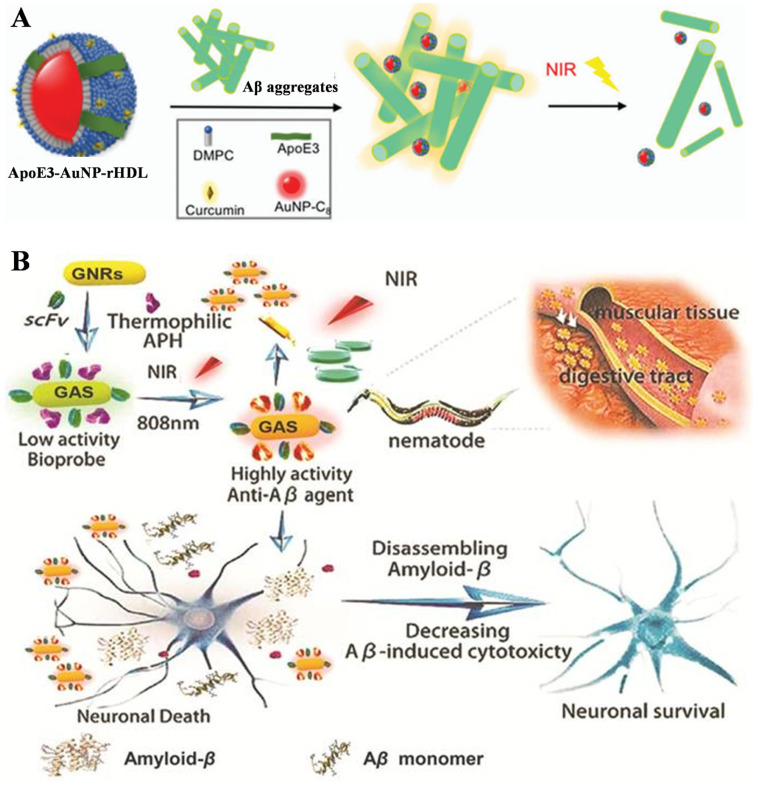
(**A**) Schematic representation of Aβ aggregate detection and light-triggered disaggregation with curcumin-loaded ApoE3–AuNP–rHDL. Reprinted with permission from ref. [68]. Copyright 2021 Royal Society of Chemistry. (**B**) Schematic illustration of GAS for AD treatment. Reprinted with permission from ref. [71]. Copyright 2019 NIH.

Metal nanoparticles have attracted the attention of scientists for over a century. They have been heavily applied in biomedical sciences and engineering. Some metal nanoparticles and their hybrid materials can inhibit Aβ aggregation and fibrillation, such as gold-based nanoparticles, nanospheres, and nanorods, as well as Pd-based nanosheets (Pd NSs) [60,61,62,63,64,65,66]. Among them, gold nanoparticles (AuNPs) have attracted increasing attention in PTT due to their easily tuned irradiation light property and inside-out hyperthermia ability [67]. Martins et al. prepared lipoprotein-based gold nanoparticles (AuNPs) for the dissociation of Aβ aggregates based on their NIR property (Figure 6A) [68]. In this work, apolipoprotein E3 (ApoE3) as a scaffold apoprotein was employed to improve the Aβ-targeting ability and BBB permeability of AuNPs. Curcumin, as a fluorescent probe was incorporated into the lipophilic domain of ApoE3 to monitor the size change of Aβ aggregates. The optical and thermal properties of gold-based nanomaterials can be precisely tuned by adjusting their shape and size [69,70]. Gold nanorods (GNRs) are ideal nanophotothermal agents because of their anisotropic shape and adjustable plasmonic properties. Liu et al. suggested that gold nanorods (GNRs) coated with single chain variable fragment (scFv) 12B4 and thermophilic acylpeptide hydrolase (APH) ST0779 as smart theranostic complexes named GAS could be used to determine and disassemble Aβ aggregates and inhibit Aβ-mediated toxicity under NIR irradiation (Figure 6B) [71]. In this method, GAS serving as both a targeting detector and an aggregation inhibitor, allowed for real-time detection of Aβ aggregates.

**Figure 7:**
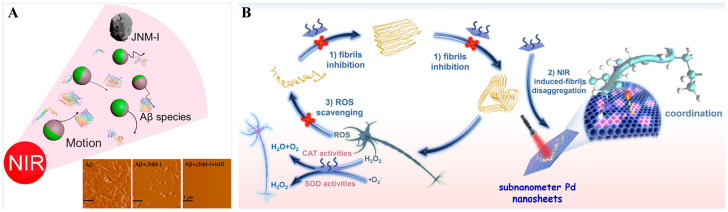
(**A**) Illustration of inhibitor-coupled JNM by conjugating Aβ-targeting peptide inhibitor D-RK10 onto the Au hemisphere of silica nanoparticles to fabricate an inhibitor-modified JNM. Reprinted with permission from ref. [72]. Copyright 2020 American Chemical Society. (**B**) Illustration of Pd NSs for inhibition of Aβ_42_ aggregation, NIR photothermal attenuation of Aβ deposition, and ROS elimination. Reprinted with permission from ref. [74] Copyright 2024 Elsevier B.V.

Nanomotors (NMs) can transfer environmental energy into mechanical motion at the nanometer/micrometer level and drive the advancement of biomedical applications. NIR light-propelled Janus NMs (JNMs) have been applied in the field of thrombus therapy, cancer treatment, deep tumor penetration, and cell membrane percolation. It has been suggested that inhibitor-modified laser-propelled JNMs (JNM-I) could modulate Aβ aggregation under NIR irradiation (Figure 7A) [72]. The nanomotor was prepared by modifying Aβ-targeting peptide DRTHLVFFARK on the Au hemisphere of SiO_2_ nanoparticles. The NIR irradiation could effectively improve the BBB penetration of JNM-I and increase the cell viability from 68% to nearly 100%. Two-dimensional metal nanosheets are receiving increasing attention due to their interesting features that differ from graphene and other inorganic nanosheets. Palladium nanosheets (Pt NSs) with strong NIR absorption have been shown to be promising photothermal agents for PTT [73]. Ding et al. found that porous Pt NSs could prevent Aβ aggregation by the Pt-Aβ interaction and disassemble Aβ fibrils by the NIR photothermal effect (Figure 7B) [74]. In addition, the Pd NSs displayed antioxidant enzyme-like activity to deplete the generated ROS and could promote cell growth with low cytotoxicity.

**Figure 8:**
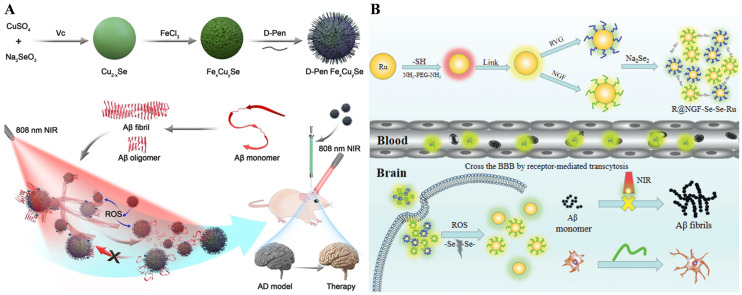
(**A**) Synthesis of penicillamine-modified FexCuySe nanoparticles and illustration of the inhibition and disassembly effects of d-Pen FexCuySe on Aβ_42_ aggregation and mitigation of potential neurotoxicity in an AD mouse model. Reprinted with permission from ref. [75]. Copyright 2020 Wiley-VCH. (**B**) Schematic illustration of the R@NGF–Se–Se–Ru nanosystem and its uptake by neuron cells. Reprinted with permission from ref. [76]. Copyright 2021 Royal Society of Chemistry.

Owing to the strong optical activity and good biocompatibility, chiral metal nanomaterials modified with chiral amino acids or peptides have received widespread attention. Zhang et al. found that chiral L/D-Fe_x_Cu_y_Se nanoparticles could modulate Aβ aggregation and disassemble Aβ fibrils into monomers under NIR irradiation (Figure 8A) [75]. The in vivo fluorescence imaging studies indicated that D-Fe_x_Cu_y_Se nanoparticles could prevent Aβ-induced neuronal damage, alleviate symptoms, and restore cognitive abilities in AD mice. Ru nanoparticles have attracted widespread attention due to their unique chemical and physical properties and good biocompatibility. Yuan et al. found that Ru nanoparticles modified with nerve growth factor (NGF) and RVG peptide could prevent Aβ aggregation by the Se–Se bonds and promote nerve regeneration (Figure 8B) [76]. NGF was used to promote neuronal regeneration for the repair of damaged nerves, and RVG peptide was used to improve BBB penetration of R@NGF–Se–Se–Ru nanoclusters. In the high ROS environment, the Se–Se bonds were broken, and small Ru nanoparticles were released from the nanoclusters. Under NIR irradiation, Aβ fibrils were disaggregated due to the good photothermal properties of Ru nanoparticles. Using NIR staining and immunoassays, the growth of nerve cells and the Aβ deposition in AD mouse brains were monitored.

#### 2.3.2. Metallic Oxides and Sulfides

**Figure 9:**
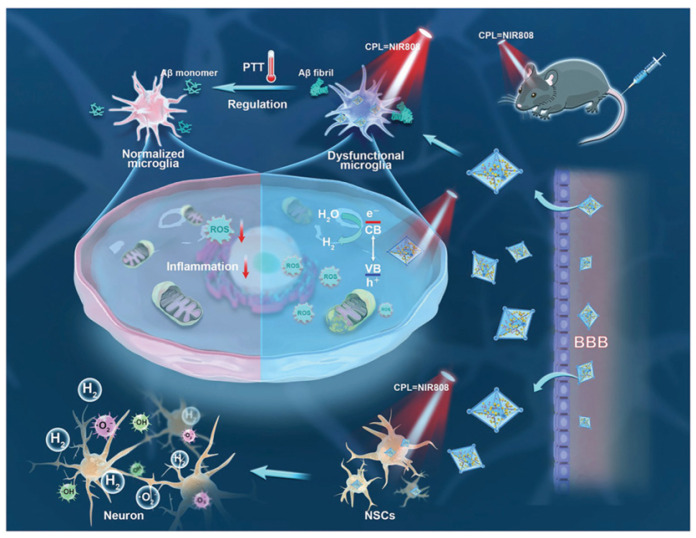
Schematic illustration of nanomaterial preparation and therapeutic mechanism in mice. Reprinted with permission from ref. [78]. Copyright 2023 Wiley-VCH.

Metallic oxides and sulfides, exhibiting both a metallic electronic structure and NIR plasmon resonance, have potential biomedical applications in imaging and diagnostics, drug delivery, and biosensor fabrication [77]. They can also modulate the aggregation of Aβ peptides, disrupt the stability of mature fibrils under NIR irradiation, and eliminate Aβ-induced ROS against neurotoxicity, typically including MoS_2_, WS_2_, and their hybrids with metal nanoparticles [78,79,80]. For instance, Tan et al. designed chiral nanomaterials UiO-66-NH_2_@L-MoS_2_ QDs@PA-Ni (denoted as MSP-U) with a triple function for AD treatment (Figure 9) [78]. The MSP-U was prepared by using UIO-66-NH_2_ as the carrier and L-Cys-modified MoS_2_ quantum dots as the central component to eliminate ROS in situ and Aβ fibrils. UIO-66-NH_2_ and doped-phytic acid Ni (PA-Ni) nanoparticles improved the activity of MoS_2_ QDs for scavenging ROS by photocatalytic H_2_ production. In addition, PA-Ni nanoparticles on the surface could stimulate neural stem cell differentiation and clear Aβ plaques. The BBB penetration of MSP-U was significantly improved by circularly polarized light (CPL) NIR irradiation, which was monitored by immunofluorescence staining experiments. The nanomaterials can be delivered into the mouse by a tail vein injection method. Their small particle size and CPL NIR facilitated the removal of nanomaterials and the decomposition of Aβ plaques. In addition, the produced H_2_ could remove ROS, protect mitochondria, and optimize the poor microenvironment of the brain, while the chiral nanomaterials could stimulate neural stem cell differentiation with CPL NIR.

**Figure 10:**
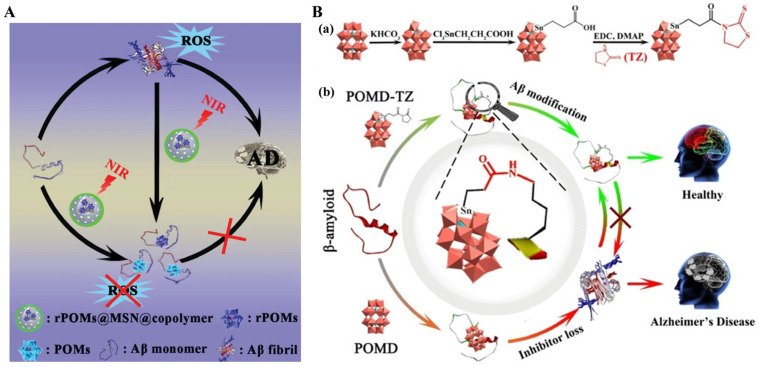
(**A**) Schematic illustration of NIR-responsive rPOMDs@MSNs@copolymer acting as a multifunctional photothermal agent for treatment of AD. Reprinted with permission from ref. [81]. Copyright 2022 Wiley-VCH. (**B**) Synthetic route to POMD-TZ (**a**). Inhibition of Aβ aggregation by modifying Aβ at the Lys_16_ site with POMD-TZ (**b**). Reprinted with permission from ref. [82]. Copyright 2022 Wiley-VCH.

Polyoxometalate (POM), a class of transition metal oxide clusters with earlier applications, shows versatile bioactivities. They can serve as effective agents to inhibit Aβ aggregation under NIR laser irradiation. Ma et al. reported a redox-activated NIR-responsive POM-based PTT material (rPOMs@MSNs@copolymer) to disaggregate Aβ fibrils and scavenge ROS (Figure 10A) [81]. This is the first report using redox-activated POM for NIR photothermal treatment of AD. In addition, a recent study suggested that the Wells–Dawson structure POM (POMD) modified with thiazolidinethione (TZ) can serve as a post-translational modification reagent to regulate amyloid aggregation (Figure 10B) [82]. The POMD-TZ functionalized with Aβ-targeted peptides and fluorescent probes has been used for AD diagnosis and therapy. POMs can inhibit Aβ aggregation, but they cannot disaggregate Aβ aggregates. Reduced POMs (rPOMs) show strong NIR absorption and good antioxidant activity. Based on this fact, Ma et al. designed an rPOMs-based photothermal material for AD treatment. It consisted of mesoporous silica nanoparticles (MSNs), rPOMs, and thermal-responsive copolymer poly(Nisopropylacrylamidecoacrylamide). The biocompatibility and bioavailability of rPOMs were increased by encapsulation in MSNs. The channels of MSNs were capped by the copolymer, limiting rPOMs leakage and improving the stability of rPOMs. Aβ fibrils could be depolymerized by the rPOMs@MSNs@copolymer via the local hyperthermia generated under NIR irradiation. Aβ-induced ROS was scavenged by the antioxidant rPOMs.

### 2.4. Others

**Figure 11:**
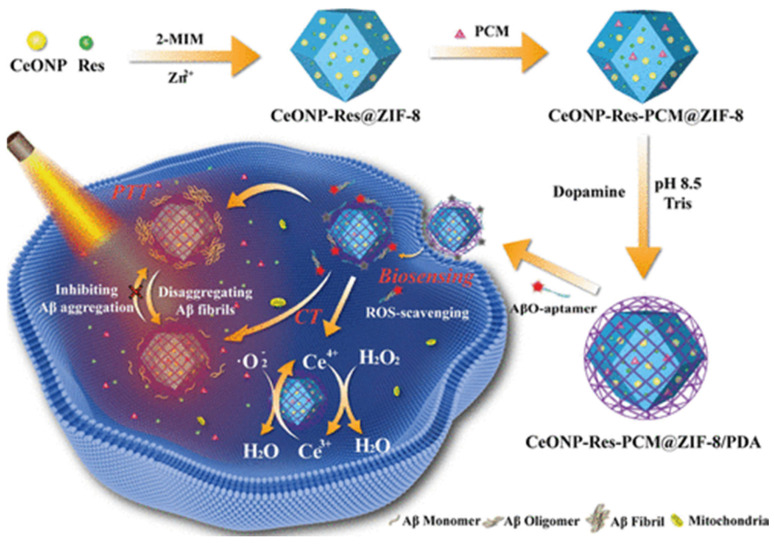
Illustration of CeONP-Res-PCM@ZIF-8/PDA preparation and its application in AβO sensing and treatment. Reprinted with permission from ref. [84]. Copyright 2021 American Chemical Society.

Inorganic-organic hybrid nanomaterials can integrate the unique advantages of both inorganic and organic elements and reduce their inherent drawbacks, demonstrating great potential in the field of PTT [83]. Yan et al. reported a multifunctional nanoagent for diagnosis and therapy of AD using ZIF-8-based materials to encapsulate resveratrol (Res), Ce nanoparticles (CeONP), and tetradecanol (PCM) (Figure 11) [84]. CeONP and Res were wrapped into ZIF-8 by the one-pot method. After the addition of PCM into CeONP-Res/ZIF-8, PDA was coated on the surface, and an aptamer specific to Aβ oligomer was modified for AD diagnosis and therapy. PCM was encapsulated in the material as an intelligent switch to achieve NIR-controlled release of therapeutic drug Res. CeONP was used as a ROS scavenger through the Ce^3+^/Ce^4+^ electron transfer. PDA served as a fluorescence quencher for the binding of aptamer and sensing of Aβ oligomer and as a photothermal agent for photothermal therapy of AD. The all-in-one system allowed the inhibition or clearance of Aβ aggregates and ROS, thereby reducing their cytotoxicity in living cells.

**Figure 12:**
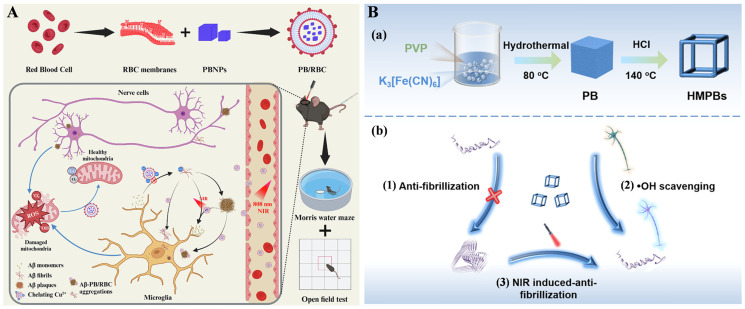
(**A**) Schematic illustration of PB/RBC synthesis and multi-target combination therapy of AD. Reprinted with permission from ref. [86]. Copyright 2024 Elsevier B.V. (**B**) Schematic illustration of the synthesis (**a**) and application (**b**) of HMPBs for inhibition of Aβ_42_ aggregation, NIR photothermal reduction of Aβ fibers, and ROS scavenging. Reprinted with permission from ref. [87]. Copyright 2025 Elsevier B.V.

Prussian blue nanoparticles (PBNPs) show good biocompatibility, stability, and high photothermal conversion efficiency under NIR irradiation [85]. Moreover, they exhibit a variety of enzyme-like activities and a high affinity for metal cations or small molecules. Li et al. found that the red blood cell (RBC) membrane-encapsulated PBNPs (PB/RBC) showed good therapeutic effect for AD (Figure 12A) [86]. In this method, PBNPs were used to chelate Cu^2+^ and remove ROS. The RBC membrane and NIR irradiation enhanced the BBB permeability of PBNPs and depolymerized the Aβ aggregates. The in vivo studies and fluorescence imaging experiments indicated that PB/RBC could improve mitochondrial quality, restore phagocytic function of microglia, alleviate neuroinflammation in AD mice, and repair memory damage. In addition, Wu et al. suggested that hollow mesocrystalline PB nanocages (HMPBs) could inhibit Aβ aggregation, depolymerize Aβ fibrils based on the NIR photothermal effect, and reduce ROS, ultimately alleviating cellular oxidative stress and improving cell survival (Figure 12B) [87].

Functionalized nanomaterials have been tailor-made in the application of PTT in non-invasive and temporo-spatial controllable AD treatment, in which the local heat enhancement can increase BBB permeability and dissolve Aβ deposits. Although PTT is still in the early stage, and high-energy lasers have non-specific adverse effects on surrounding healthy tissue, this strategy is of great significance in AD phototherapy, especially when combined with other therapies such as immuno/chemo synergic therapy. It is meaningful to synthesize PTT nanomaterials for the dissociation of Aβ aggregates and the release of Aβ-targeted drugs under the irradiation of low-energy light.

## 3. PDT Methods

The primary component of PDT is the photosensitizer, a light-sensitive molecule that can selectively localize within target cells and/or tissues [88,89]. Upon activation by light in the presence of oxygen, the photosensitizer will undergo a photochemical reaction, generating various ROS, including HO•, O_2_•^−^, and ^1^O_2_. These ROS can react with amyloid peptides, initiating the oxidation of specific residues such as methionine (Met), histidine (His), and tyrosine (Tyr). This oxidative modification can enhance the hydrophilicity of Aβ, thereby inhibiting its fragmentation and degradation and suppressing Aβ-induced cytotoxicity. The PDT materials used for AD treatment mainly include organic molecules, metal complexes, nanomaterials, and upconversion nanoparticles (UCNPs).

### 3.1. Small Organic Molecules

Photosensitizers of small organic molecules are now being widely applied for PDT in the clinic or undergoing evaluation in clinical trials [90,91,92,93,94]. Some of them were used to identify and treat AD by imaging and light-triggered anti-aggregation or degradation of Aβ through photodynamic reactions, including thioflavin-T (ThT) derivatives [95,96,97,98,99], fullerenes [100], heterocyclic compounds (e.g., riboflavin vitamin B2, methylene blue or MB, rose bengal or RB) [101,102,103,104,105], porphyrin derivatives [106,107,108], quinoline derivatives [109,110,111,112], curcumin derivatives [113], boron-dipyrromethene (BODIPY) [114], azobenzene–boron complexes [115,116], and anthraquinone series [117], as shown in Table 1. In this section, we briefly discuss the progress in the diagnosis and treatment of AD with small organic molecules as PDT photosensitizers. Only several important and typical works discussed in detail as examples, as interested researchers can find the detailed information in the previously reported impressive review papers [30,90].

**Figure 13:**
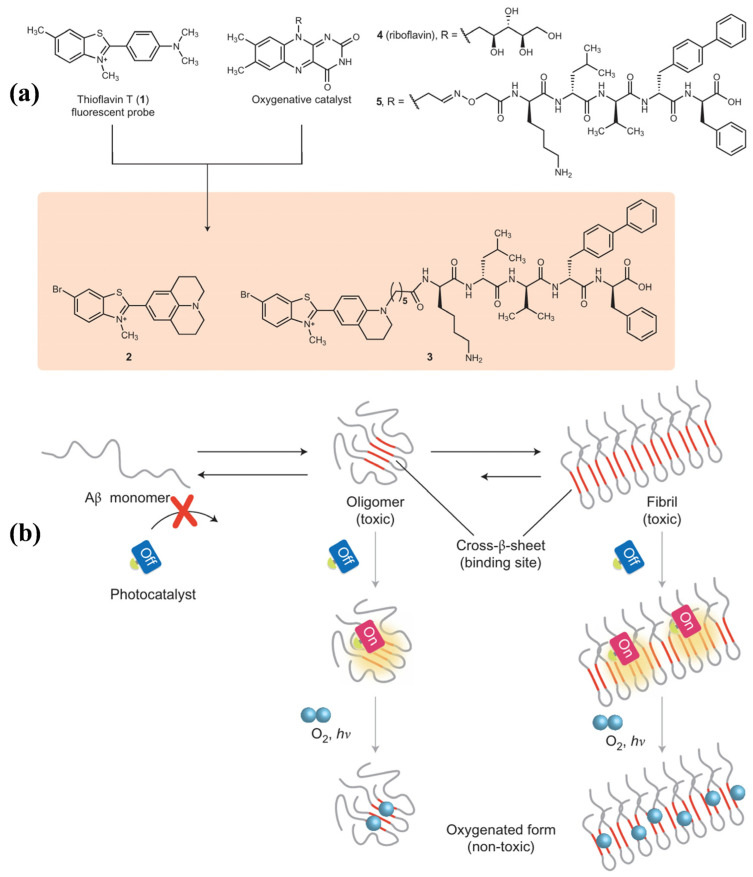
(**a**) Molecular design of cross-β-sheet-sensing photooxygenation catalysts 2 and 3. (**b**) Conceptual scheme of the oxygenation mechanism of the on/off switchable photooxygenation catalyst devised with the TaSCAc approach. Reprinted with permission from ref. [96]. Copyright 2016 Springer Nature.

**Figure 14:**
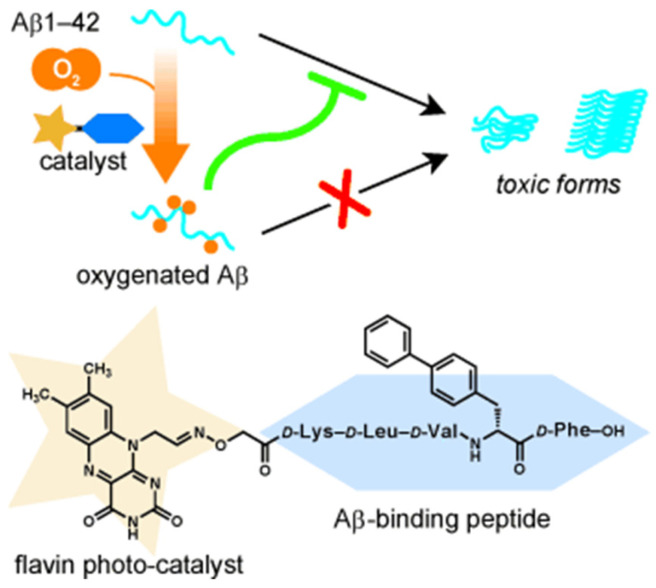
Schematic illustration for riboflavin-catalyzed photooxygenation of Aβ occurred in well-defined positions under physiologically relevant conditions. Reprinted with permission from ref. [101]. Copyright 2014 Wiley-VCH.

**Figure 15:**
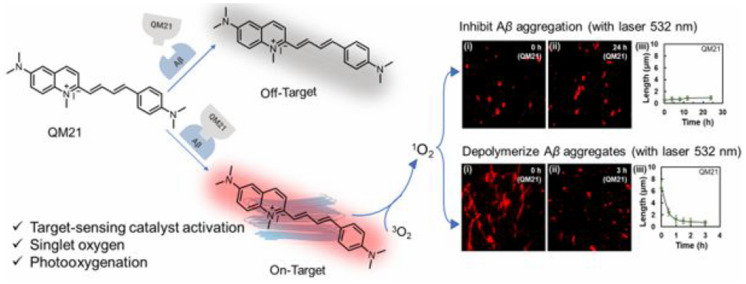
Chemical structure of photosensitizers and confocal light microscopy images for photooxygenation of Aβ monomers or fibrils stained with QM21 under laser irradiation at 0 (**i**) and 24 h (**ii**). (**iii**) Corresponding length of Aβ monomers or fibrils stained with QM21 under laser irradiation for different time. Reprinted with permission from ref. [110]. Copyright 2022 American Chemical Society.

ThT is a well-known fluorescence probe for recognizing Aβ aggregates. Since it was found that ThT could produce ^1^O_2_ and disrupt preformed Aβ fibrils under laser irradiation [95], a series of ThT derivatives have been designed and used for Aβ imaging and photooxidation. For example, Taniguchi et al. developed a target-state-dependent photooxygenation catalyst for selectively oxygenating pathogenic aggregated amyloid proteins through a target-sensing catalyst activation (TaSCAc) approach (Figure 13) [96]. In this method, the activity of catalyst was turned on only when it bound to a specific target protein structure. The catalyst can be activated only when binding to the cross-β-sheet structures of amyloidogenic proteins, such as Aβ, amylin, insulin, β2-microglobulin, transthyretin, and α-synuclein. N- or O-heterocyclic compounds show high triplet quantum yields, low phototoxicity, and low cytotoxicity under dark conditions, making them suitable for biomedical applications. Taniguchi et al. also suggested that the Tyr^10^, His^13^, His^14^, and Met^35^ residues in Aβ can be photooxygenated by a riboflavin catalyst with visible light irradiation, inhibiting the aggregation of Aβ and reducing its neurotoxicity (Figure 14) [101]. In addition, Yang et al. reported a series of D-π-A photosensitizers based on a quinolinium scaffold (electron acceptor) and a dimethylaniline group (electron donor) (Figure 15) [110]. The length of the vinyl group (π-bridge) was mediated via the TaSCAc strategy. The N,N-dimethylamino moiety could bind Aβ aggregates, thereby turning on the NIR emission and enhancing the production of ^1^O_2_ by inhibiting the intramolecular rotation and twisted-intramolecular charge transfer process. The turn-on fluorescence and the generated ^1^O_2_ allowed for the specific imaging and photo-oxygenation of Aβ aggregates, clearing Aβ aggregates, and reducing neurotoxicity via the microglial lysosomal pathway.

**Table 1:** Small organic photosensitizers for modulating Aβ aggregation by the PDT method.

Photosensitizers	Ex. (nm)	Application	Ref.
ThT	442	Degrading Aβ40 fibrils under light	[95]
ThT derivative	500	Targeting Aβ42 aggregates to reduce PC12 cytotoxicity	[96]
ThT derivative	LED light	Photooxidation of Tyr10, His13, His14, and Met35 in Aβ42	[97]
ThT derivative	450	Degrading Aβ42 aggregates	[99]
Fullerene	365	Inhibiting Aβ42-mediated PC12 cytotoxicity	[100]
Riboflavin T	White	Aβ42-mediated PC12 cytotoxicity by oxidation of Tyr10, His13, His14, and Met35	[101]
Methylene blue	630	Decomposition of Aβ42 aggregates to reduce drosophila cytotoxicity	[102]
Rose bengal	525	Inhibiting Aβ42-mediated PC12 cytotoxicity	[103]
1,2,4-Oxadiazole	260	Inhibiting Aβ40-mediated LAN-5 cytotoxicity	[105]
Porphyrin derivative	365	Inhibiting Aβ42-mediated PC12 cytotoxicity	[106]
Porphyrin derivative	450	Inhibiting neurodegenerative manifestations in AD Drosophila	[108]
Chlorin e6	Visible light	Inhibiting Aβ40-mediated PC12 cytotoxicity	[107]
Quinoline derivatives	614~830	Degrading Aβ plaques in AD mouse brain	[109]
Donor-π-Acceptor	561	Photooxygenation of Aβ40 aggregates in PC12 cells	[110]
Donor-π-Acceptor	635	Photooxygenation of Aβ40 aggregates in PC12 cells	[111]
Donor-π-Acceptor	520	Photooxygenation of Aβ42 aggregates in SH-SY5Y cells	[112]
CRANAD	780	Degrading Aβ aggregation in an AD mouse model	[113]
Azobenzene-boron	595	Degrading brain Aβ42 in AD mice	[115]
Leuco ethyl violet	595	Oxygenating Aβ in vivo	[116]
Anthraquinone series	467	Controlling the pathological factors of AD	[117]

**Figure 16:**
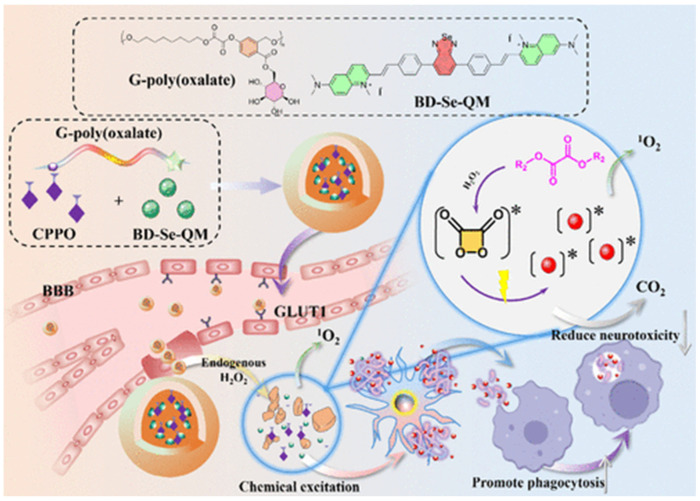
Schematic diagram of the synthesis process of BD-Se-QM/NPs and the in situ chemical excitation of BD-Se-QM/NPs by H_2_O_2_ to oxidize Aβ_1–42_ aggregates, promoting uptake by microglial cells. Reprinted with permission from ref. [118]. Copyright 2024 American Chemical Society.

In addition, considering that the phototherapy strategy has the disadvantage of limited penetration of the external light source into deep tissue, Liu et al. reported a chemiluminescence-initiated photodynamic therapy strategy instead of external laser irradiation (Figure 16) [118]. It consists of D-glucose-based polyoxalate G-poly(oxalate), photosensitizer BD-Se-QM, and bis[2,4,5-trichloro 6-(pentoxy-carbonyl)phenyl]ester. The photosensitizer has a good ability to image Aβ plaques, generate ^1^O_2_, and photooxidize Aβ aggregates under white light. In this method, G-poly(oxalate) was used to enhance the BBB permeability and chemiluminescence resonance energy transfer efficiency. The oxalate ester groups can promote the production of high-energy intermediates in the presence of H_2_O_2_, thereby activating BD-Se-QM to generate ^1^O_2_ and inhibit Aβ aggregates, promoting microglial Aβ uptake, and reducing Aβ-induced neurotoxicity. In addition, other organic hybrid materials have also been used for the photo-oxidation of Aβ fibrils, such as oligomeric p-phenylene ethynylenes [119] and corannulene (Cor)-conjugated rhodamine B isothiocyanate (Rhb) [120].

### 3.2. Metal Complexes

**Figure 17:**
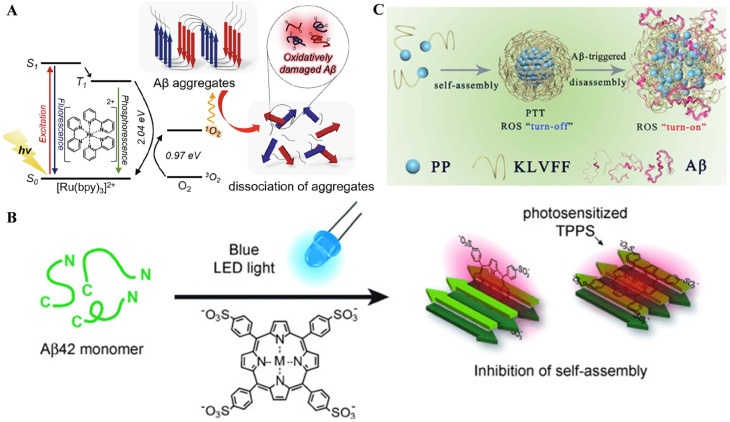
(**A**) Schematic illustration of the dissociation of Aβ aggregates through oxidative damage by photoexcited [Ru(bpy)_3_]^2+^. Reproduced with permission from ref. [124]. Copyright 2021 Elsevier B.V. (**B**) Diagram for inhibition of Aβ self-assembly into fibril by blue LED light-sensitized TPPS. Reproduced with permission from ref. [108]. Copyright 2016 Wiley-VCH. (**C**) Schematic illustration of the self-assembly process and Aβ-triggered disassembly process of PKNPs. Reproduced with permission from ref. [125]. Copyright 2023 Royal Society of Chemistry.

As a class of chemical modulators against Aβ aggregation, metal complexes have been designed and used to target and regulate Aβ peptides through coordination chemistry and oxidative and proteolytic reactions based on their tunable properties, including the oxidation state and coordination geometry of metal centers such as Ru(II) and Ir(III)) [121,122,123]. For example, Park and co-workers found that the photoactive tris(2,20-bipyridine)ruthenium (II) ([Ru(bpy)_3_]^2+^) could promote the photodissociation of Aβ aggregates into small, non-toxic fragments by oxidative damage (Figure 17A) [124]. Porphyrins are a type of organic photosensitizer usually existing in the form of coordination with metal ions. Lee et al. found that the photoactivated meso-tetra(4-sulfonatophenyl)porphyrin (TPPS with M = 2H^+^, Zn^2+^, Cu^2+^, Mn^2+^) could bind Aβ and inhibit its aggregation in vitro by photooxidation (Figure 17B) [108]. The effect on photoinduced inhibition of Aβ aggregation and its toxicity was dependent on the metal ions coordinated with porphyrins. It was also found that TPPS could suppress synaptic toxicity, neural cell death, and behavioral defects in the Drosophila AD model under blue light illumination. However, these photosensitizers cannot simultaneously achieve enhanced BBB permeability and selective photooxygenation of Aβ, resulting in poor therapeutic efficacy, serious off-target toxicity, and substandard bioavailability. From this perspective, Liu et al. designed target-driven supramolecular self-assemblies named PKNPs to prevent Aβ aggregation in vivo with enhanced BBB penetrability and switchable photoactivity (Figure 17C) [125]. The PKNPs were prepared by the self-assembly of Aβ-targeting peptide (KLVFF) conjugated with a porphyrin derivative (5-(4-carboxyphenyl)-10,15,20-triphenylporphyrin). The BBB permeability of PKNPs was 8.5-fold higher than that of porphyrin alone due to the photothermal effect. After binding with Aβ, the morphology of PKNPs changed from a sphere into an amorphism form. The disassembled PKNPs could selectively oxygenate Aβ by the transformation of photothermal activity into photodynamic activity.

**Figure 18:**
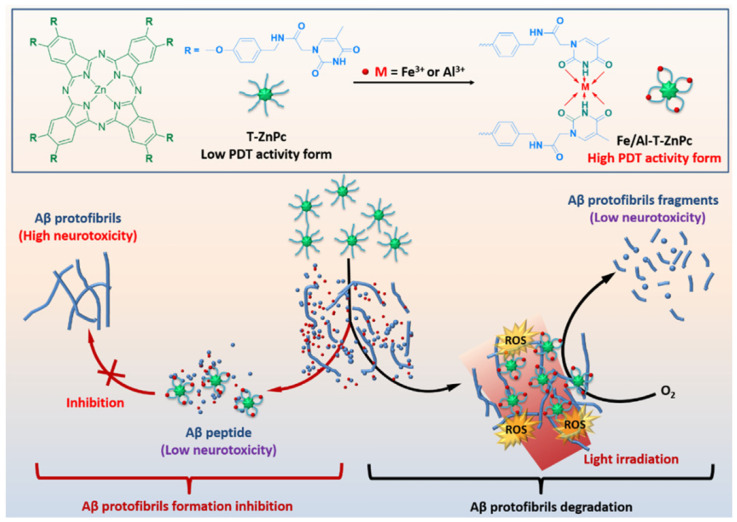
Structure of T-ZnPc and mechanism illustration for photodegradation of Aβ fibrillation and Aβ peptide aggregation inhibition in the presence of Fe^3+^ or Al^3+^. Reprinted with permission from ref. [127]. Copyright 2019 Elsevier B.V.

Phthalocyanine (PC) is another photosensitizer widely used in PDT due to its high extinction coefficient, long absorption wavelength, and adjustable photophysical and photochemical properties [126]. Relatively high levels of Al^3+^ and Fe^3+^ were found around Aβ aggregates in the AD brain. Zhan et al. suggested that thymine-modified Zn phthalocyanine (T-ZnPc) can be coordinated with Al^3+^ and Fe^3+^ to activate the PDT activity (Figure 18) [127]. The generated ROS by Al-T-ZnPc and Fe-T-ZnPc degraded Aβ protofibrils and reduced neurotoxicity at a degradation of 62% and 81%, respectively. In addition, T-ZnPc could inhibit the formation of Aβ protofibrils and chelate free Al^3+^ and Fe^3+^ in the brain.

### 3.3. Nanomaterials

#### 3.3.1. Nanodots

**Figure 19:**
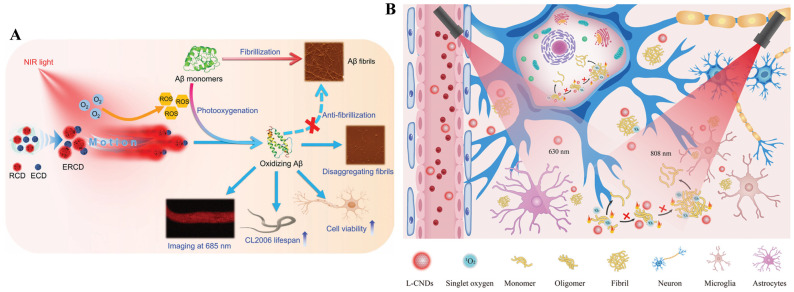
(**A**) Schematic illustration of the multifunctionality of ERCD. Reprinted with permission from ref. [130]. Copyright 2024 Wiley-VCH. (**B**). Photooxidative and photothermal carbon nanodots for inhibiting amyloid aggregates and alleviating Aβ_42_-induced cytotoxicity. Reprinted with permission from ref. [132]. Copyright 2025 Elsevier B.V.

With the tremendous development of nanotechnology, various functional nanomaterials have shown application potential in the biomedical field, such as drug delivery, anti-tumor, and antibacterial therapy. They can enhance the stability and selectivity of drug loading, reduce drug-induced side effects, achieve controlled drug release and targeted delivery, and improve therapeutic efficacy. Based on their unique physical, chemical, and optical properties, nanomaterials can also be used as photosensitizer carriers or direct photosensitizers, significantly improving the efficacy and safety of PDT [128]. Recently, photoresponsive nanomaterials have been well developed and applied for the diagnosis and treatment of AD by modulating Aβ aggregation [129,130,131,132]. For example, Xu et al. investigated the photo-triggered inhibition of Aβ aggregation and cytotoxicity of polymer nanodots (Pdots) modified with RB, MB, and riboflavin based on the formation of ROS, including ^1^O_2_ [133]. Among these Pdots, RB-Pdots showed better biocompatibility and a higher ability for ^1^O_2_ production. Lin et al. developed multifunctional dual-carbon dots named ERCD composites with photo-propelled nanomotor behavior for AD treatment (Figure 19A) [130]. The composites consist of near-infrared CDs (RCD) and epigallocatechin gallate-derived carbonized polymer dots (ECD). The light-driven Janus nanomotors could image Aβ plaques in vivo at 685 nm and rapidly disaggregate mature Aβ fibrils by photooxygenation under NIR irradiation, ultimately reducing Aβ-induced cytotoxicity and prolonging the lifespan of AD nematodes by 6 d at 2 μg/mL. In addition, Shao et al. found that light-sensitive carbon nanodots (L-CNDs) exhibited good photocatalytic properties for the production of ^1^O_2_ under 630 nm irradiation (Figure 19B) [132]. The inhibition efficiency for Aβ aggregation reached 61.08%, 75.09%, and 91.72% by PTT, PDT, and synergistic PTT/PDT, respectively. The L-CNDs, with a photothermal conversion efficiency of 68.25% under 808 nm irradiation produced local heat, regulating Aβ aggregation and mitigating Aβ-induced cytotoxicity in PC12 and HT22 cells.

#### 3.3.2. Two-Dimensional Nanomaterials

**Figure 20:**
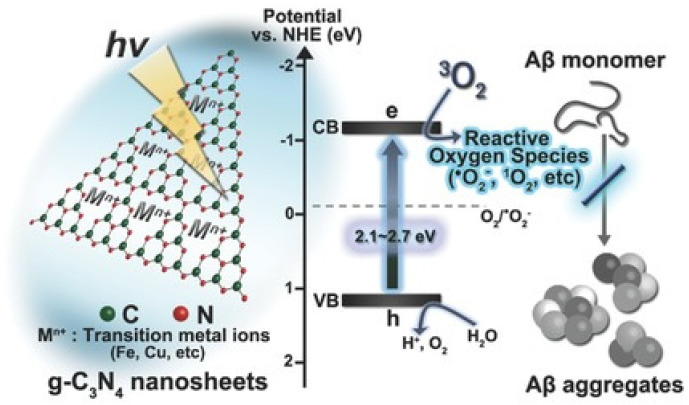
Schematic illustration of g-C_3_N_4_ nanosheets as Aβ aggregation inhibitors. Reprinted with permission from ref. [135]. Copyright 2016 Wiley-VCH.

Two-dimensional semiconductors offer unique advantages in the aspects of biocompatibility, stability, and chemical enhancement capability. Among the potential two-dimensional semiconductors, graphitic carbon nitride (g-C_3_N_4_) stands out for its excellent electron conductivity and high adsorption capacity [134]. g-C_3_N_4_ materials have been designed to bind Aβ and modulate its aggregation and toxicity. For example, Park and co-workers showed that g-C_3_N_4_ nanosheets could inhibit Aβ aggregation and toxicity (Figure 20) [135]. Under visible-light illumination, the exfoliated g-C_3_N_4_ nanosheets promoted the generation of ROS (e.g., superoxide anion and singlet oxygen) by photoinduced electron transfer. Doping transition metal ions in g-C_3_N_4_ nanosheets further accelerated the charge transfer, generating more ROS species and improving inhibition efficiency toward Aβ aggregation.

**Figure 21:**
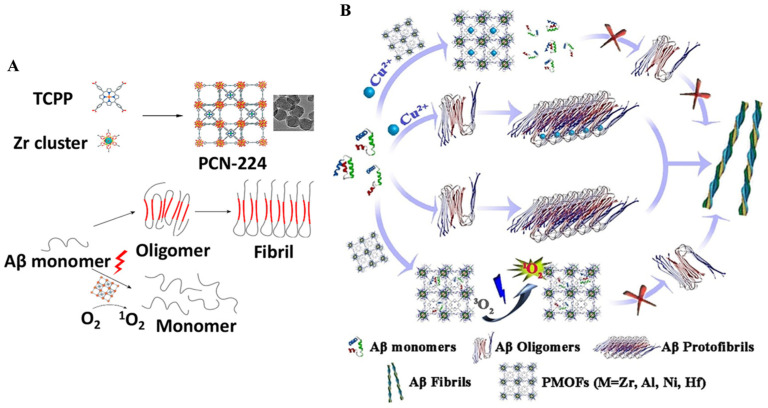
(**A**) Schematic illustration of photo-inhibition of Aβ_42_ aggregation by PCN-224 nanoparticles. Reprinted with permission from ref. [136]. Copyright 2018 American Chemical Society. (**B**) Schematic illustration of the inhibition effect of PMOFs on the amyloid fibrillation process. Reprinted with permission from ref. [137]. Copyright 2019 Wiley-VCH.

Due to the multifunctional building units and the potential for modification and engineering after synthesis, MOFs have attracted the widespread interest of researchers. Using MOFs as precursors or sacrificial templates, various multifunctional nanomaterials derived from MOFs can be produced and used in various biomedical fields. MOF PCN 224 nanoparticles can be hydrothermally synthesized by the coordination of tetra-kis(4-carboxyphenyl)porphyrin (TCPP) and zirconium. Wang et al. suggested that the porphyrinic PCN-224 nanoparticles, which exhibit high photo-oxygenation efficiency, good biocompatibility, and high stability could suppress Aβ aggregation and reduce Aβ-induced cytotoxicity under NIR irradiation (Figure 21A) [136]. In addition, Yu et al. investigated the ability of four porphyrinic MOFs (PMOFs) including Zr–MOF, Al MOF, Ni–MOF, and Hf–MOF to chelate Cu^2+^ and inhibit Aβ aggregation by photooxidation (Figure 21B) [137]. Among them, Hf–MOF showed the highest photooxidation efficiency for the generation of ^1^O_2_. The modification of Aβ-targeting peptide LPFFD on the Hf–MOFs enhanced decreased Aβ-induced cytotoxicity and promoted Aβ photooxidation in the transgenic AD model. In this work, thioflavin S (ThS) staining was used to monitor the changes in Aβ levels in the head region of worms by fluorescent imaging.

**Figure 22:**
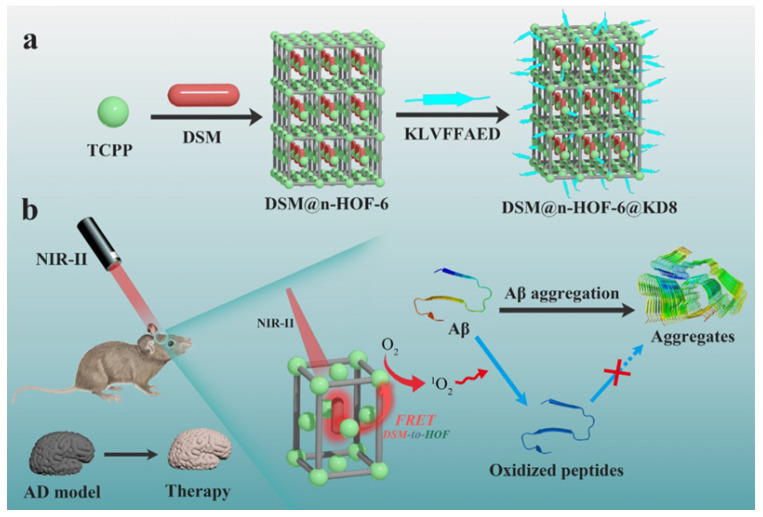
(**a**) Schematic illustration of NIR-II HOFs synthesis. (**b**) Representation of inhibitory effect mechanism on Aβ aggregation and mitigation of potential neurotoxicity in AD model mice. Reprinted with permission from ref. [140]. Copyright 2022 Wiley-VCH.

As a novel type of porous crystalline material, hydrogen-bonded organic frameworks (HOFs) can be constructed from organic or metal-organic building blocks through hydrogen bonding interactions [138,139]. Zhang et al. used meso-tetrakis(carboxy phenyl)porphyrin (TCPP)-based HOFs to encapsulate pyridinium hemicyanine dye 4-[p-(dimethyla mino)styryl]-1-methylpyridinium) (DSM) with a large two-photon absorption (TPA) cross-section in the NIR-II window (Figure 22) [140]. Aβ-targeted peptide KLVFFAED (KD8) was modified on DSM@n-HOF-6 to enhance the BBB permeability. The resulting DSM@n-HOF-6@KD8 exhibited two-photon NIR-II-excited fluorescence resonance energy transfer (FRET) to promote the generation of ^1^O_2_ for Aβ oxidation and good biocompatibility in rat pheochromocytoma (PC-12) cells. They could reduce craniocerebral Aβ plaques and Aβ-mediated cytotoxicity and improve cognitive ability in AD model mice.

### 3.4. UCNPs

**Figure 23:**
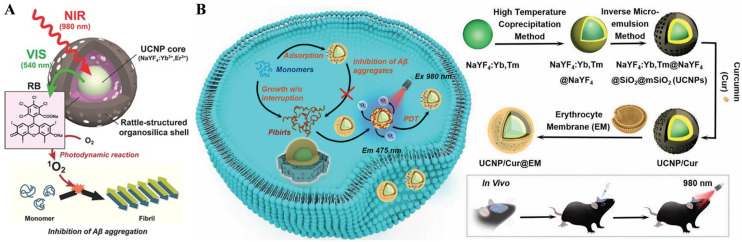
(**A**) Schematic illustration of Aβ aggregation inhibition by NIR-mediated rattle-structured UCNPs loaded with RB. Reprinted with permission from ref. [145]. Copyright 2017 Wiley-VCH. (**B**) Schematic illustration of biomimetic UCNP-based PDT for the inhibition of Aβ aggregates. Reprinted with permission from ref. [146]. Copyright 2023 Wiley-VCH.

UCNPs are a promising new generation of biological imaging reagents. Under NIR light excitation, UCNPs can emit high-energy visible light for activating the surrounding photosensitizer molecules to produce ^1^O_2_. Compared to traditional PDT induced by visible or ultraviolet light, NIR-excited UCNPs can be used to activate photosensitizer molecules in deeper tissues because of their high tissue penetration ability of NIR light. UCNPs-based PDT methods have been developed recently for AD treatment by modulating Aβ aggregation [141,142,143,144]. For example, Kuk et al. used RB-loaded NaYF_4_:Yb,Er UCNPs to inhibit Aβ aggregation by the generated ^1^O_2_ (Figure 23A) [145]. An organosilica shell was deposited on the UCNPs to improve the energy transfer efficiency for the disaggregation of RB. The emitted visible light (540 and 660 nm) was absorbed by the loaded RB. The photoexcited RB promoted the generation of ^1^O_2_ through photodynamic reaction to suppress Aβ aggregation. Furthermore, Wang et al. proposed erythrocyte membrane (EM)-modified core–shell Yb/Er-co-doped NaYF4 (NaYF4:Yb,Er) UCNPs for improving the PDT efficiency in AD treatment (Figure 23B) [146]. Photosensitizer curcumin (Cur) was loaded in the UCNPs with a mesoporous silica layer to generate ROS under 980 nm NIR irradiation. In this work, EM was used to enhance the Aβ-binding ability, restrain Aβ aggregation, and promote the degradation of Aβ aggregates by ROS. The in vivo experiments indicated that the UCNP/Cur@EM could decrease Aβ deposits, ameliorate memory deficits, and rescue cognitive function in transgenic mice.

During the continuous development of photodynamic AD therapy, scientists around the world have made tremendous efforts to explore novel photosensitizers and PDT strategies for inhibiting Aβ aggregation and decomposing Aβ fibers. Due to its excellent photo-oxygenation effect, high temporo-spatial controllability, and minimized side effects, PDT has become an important candidate for AD treatment, although its efficiency heavily depends on the O_2_ content in the surrounding environment. Therefore, it is necessary to develop nanomaterials for the continuous generation of O_2_ to overcome the problem of O_2_ deficiency and improve the PDT efficacy.

## 4. AIE-Based Strategies for Imaging and Phototherapy of AD

**Figure 24:**
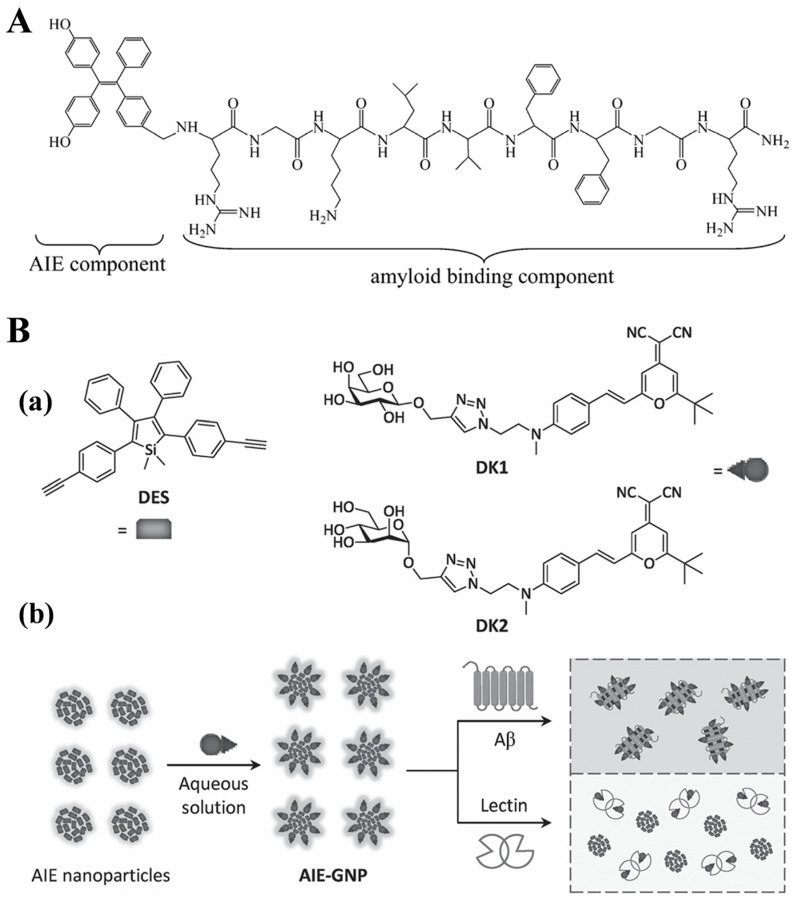
(**A**) Structure of the fluorescent probe showing AIE and amyloid binding peptide components. Reprinted with permission from ref. [162]. Copyright 2015 American Chemical Society. (**B**) (**a**) Structures of the AIEgen (DES) and glycoprobes (DK1 and DK2). (**b**) Schematic illustration of the supramolecular assembly of AIEgen with glycoprobe to produce the AIE-GNP for ratiometric detection of Aβ and discrimination with lectin. Reprinted with permission from ref. [163]. Copyright 2016 Wiley-VCH.

The commercial thioflavin derivatives for in vivo imaging of Aβ plaques, such as ThT or ThS, are limited by small Stokes shift, low signal-to-noise ratio, and poor BBB penetrability [147]. As a novel type of advanced material, AIEgens have been developed and applied in various fields with excellent performances [148,149,150,151]. Compared with traditional fluorophores with aggregation-caused quenching effects, AIEgens have strong NIR deep penetration, large Stokes shifts, excellent biocompatibility and photostability, and desirable BBB permeability. In this regard, AIE materials have been designed to image Aβ plaques and modulate Aβ aggregation for the diagnosis and treatment of AD, including quinoline-based AIE probes [152], DM-V2CN-PYC3 [153,154], PD-NA-TEG [155], Cou-AIE-TPP^+^ [156], AIE-CNPy-AD [157], ASCP [158], TPE-TPP [159], crystal violet [160], and pyrene-based dyes [161], as shown in Table 2. In this section, only several important and typical examples were discussed, as the detailed information of others can be found in the previous reviews [148,150]. For example, Pradhan et al. reported an AIE-based probe for the specific and reliable detection of amyloid fibrillation (Figure 24A) [162]. The probe contains a TPE-based AIEgen and an RGKLVFFGR-based amyloid-binding peptide. Compared to the commonly used ThT dye and other probes for amyloid fibrils, the AIE probe showed a high signal-to-noise ratio and specificity toward Aβ fibrils, insulin fibrils, and lysozyme fibrils due to the selective binding of RGKLVFFGR to the amyloid structure. In addition, He’s group reported a ratiometric AIE glyconanoparticle (AIE-GNP) used for the detection of Aβ peptides and fibrils (Figure 24B) [163]. The nanoparticle was prepared by the supramolecular assembly of a silole-based AIEgen and a glycoprobe. This work provides insights into the development of novel multifunctional probes for ratiometric detection of biomolecules based on AIEgens and other materials.

**Figure 25:**
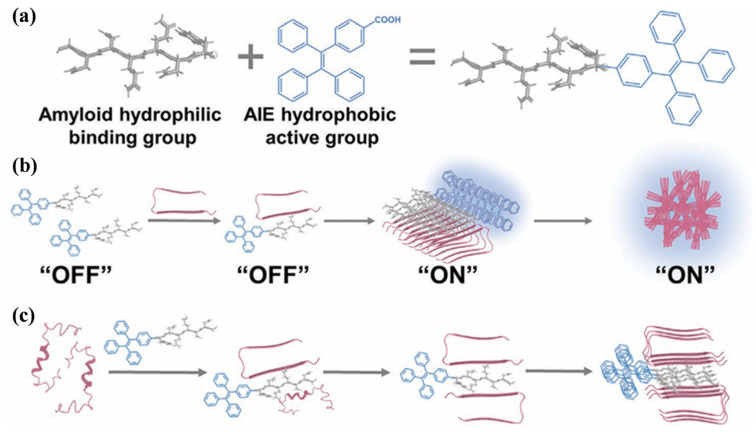
(**a**) A “step-by-step” strategy to construct G7-TBA by conjugating a GNNQQNY from yeast prion protein Sup35 and an AIE-active TBA. (**b**) G7-TBA as an “off-on” probe for amyloid detection. (**c**) G7-TBA as an amyloid modulator to accelerate amyloid fibrillization. Reprinted with permission from ref. [164]. Copyright 2022 Wiley-VCH.

Tang et al. synthesized an AIE-active molecule through the conjugation of an amyloid fragment (GNNQQNY, named G7) with triphenyl vinyl benzoic acid (TBA) (Figure 25) [164]. The G7-TBA conjugate was used as both probe and modulator to determine, monitor, and alter the aggregation of three different amyloid proteins (Aβ, hIAPP, and hCT). The bifunctional molecule showed conformationally specific binding affinity for amyloid aggregates, switching from the “off” state for amyloid monomers to the “on” state for β-structure-rich amyloid oligomers and fibrils. As a kind of amyloid modulator, G7-TBA accelerated amyloid fibrillization and selectively protected cells from hIAPP-induced toxicity. The binding affinity between G7 and amyloid aggregates is higher than that of ThT. This concept is valuable for the design of amyloid-AIE conjugates as multifunctional probes and modulators for biomedical applications.

**Figure 26:**
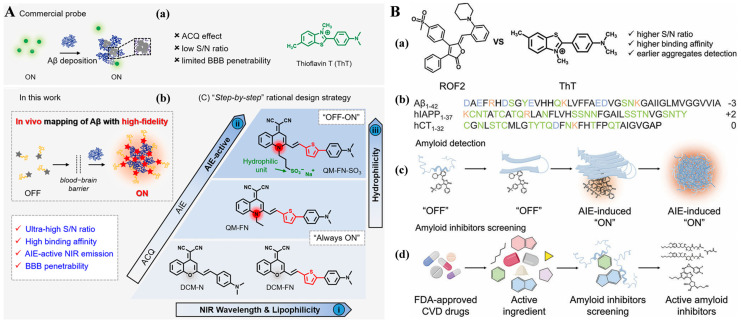
(**A**) Rational design of NIR AIE-active probes for Aβ deposition. (**a**) Commercial probe ThT based on the always-on pattern. (**b**,**c**) The “step-by-step” strategy to address the inherent defects of commercial ThT and create ultrasensitive off-on NIR probes. Reprinted with permission from ref. [165]. Copyright 2019 American Chemical Society. (**B**) Dual-functional ROF2 fluorescence for amyloid detection and amyloid inhibitor screening. (**a**) Chemical structure of ROF2 and ThT. (**b**) Aβ, hIAPP, and hCT sequences. (**c**) “Off-on” switch detection of amyloid aggregates with ROF2 as an amyloid probe. (**d**) Screening of amyloid inhibitors using ROF2. Reprinted with permission from ref. [167]. Copyright 2024 Wiley-VCH.

The unwanted initial aggregation of AIEgens before binding to Aβ aggregates may lead to “false-positive” signals. In order to overcome the dilemma between the requirement of lipophilicity for long-term emission and aggregation behavior from water to protein fibrils, Fu et al. designed an AIE-active NIR probe named QM-FN-SO_3_ as an effective alternative to ThT or ThS for in vivo imaging of Aβ plaques [165,166]. A lipophilic π-conjugated thiophene-bridge was included in the probe to extend the wavelength in the NIR region and enhance the BBB penetrability (Figure 26A). The AIE probe was designed based on the ACQ building block. The sulfonate group guaranteed specific hydrophilicity of the probe and maintained it at the signal-off state before binding to Aβ plaques. The probe could eliminate both the self-quenching distorted signal from ThT and the “false-positive” signal from initial aggregation, thus meeting the high-fidelity requirement to image Aβ plaques in vivo. This work, for the first time, solved the dilemma between the lipophilicity requirement of NIR emission and the aggregation behavior from water to protein fibrillogenesis, achieving a breakthrough in high-fidelity detection of Aβ plaques. In addition, Zheng’s group reported a multifunctional AIEgen called ROF2 as the amyloid probe and inhibitor (Figure 26B) [167]. Compared to ThT, ROF2 exhibited superior sensing capability in monitoring, imaging, and distinguishing amyloid aggregates with different sequences and sizes. ROF2 as an imaging reagent showed excellent amyloid inhibition ability, efficiently preventing amyloid aggregation and reducing amyloid-induced cytotoxicity both in cells and nematodes.

**Figure 27:**
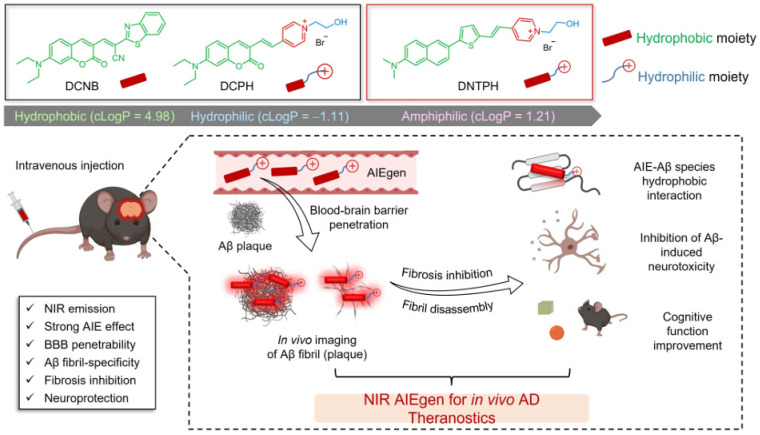
Design of NIR AIEgens via a balanced hydrophobicity-hydrophilicity strategy. The multi-optimized AIEgen called DNTPH for in vivo AD theranostics shows BBB penetration, Aβ fibrosis inhibition, and recovery of cognitive function in AD mice. Reprinted with permission from ref. [168]. Copyright 2023 Wiley-VCH.

Recently, Zhang et al. reported a concept for designing NIR AIEgens as the theranostic agents to image Aβ plaques through a balanced hydrophobicity-hydrophilicity method (Figure 27) [168,169]. The designed multifunctional AIEgen called DNTPH could bind Aβ fibrils with a high signal-to-noise ratio and selectivity. The in vivo imaging experiments proved its excellent BBB permeability and long-term tracking ability for AD diagnosis. The learning deficit in AD mice was rescued after DNTPH treatment. Overall, the AIEgen probe exhibited a strong ability to inhibit Aβ aggregation, promote fibril disassembly, and ultimately attenuate Aβ-induced neurotoxicity. This is the first report using AIEgen as a theranostic agent for in vivo real-time NIR imaging of Aβ plaques and AD therapy simultaneously.

**Figure 28:**
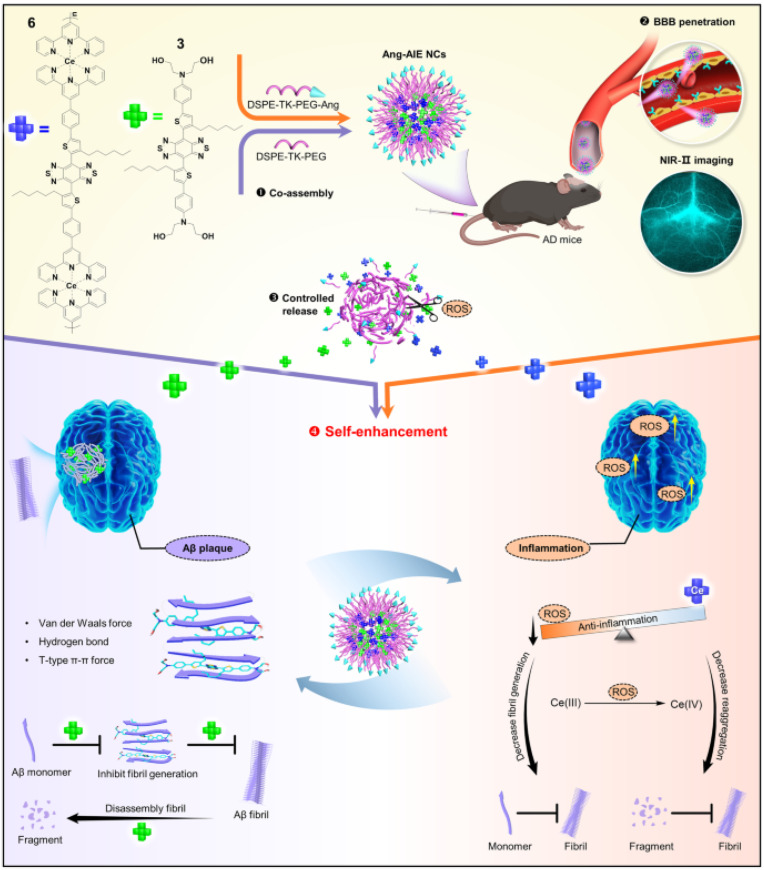
Schematic illustration of NIR-II brain-targeted theranostic system for dual-target therapy of AD by co-assembly of nanodrug, NIR-II imaging, inflammation-associated ROS environment, and activation of the self-enhanced dual-targeting program. Reprinted with permission from ref. [170]. Copyright 2024 Springer Nature Limited.

**Figure 29:**
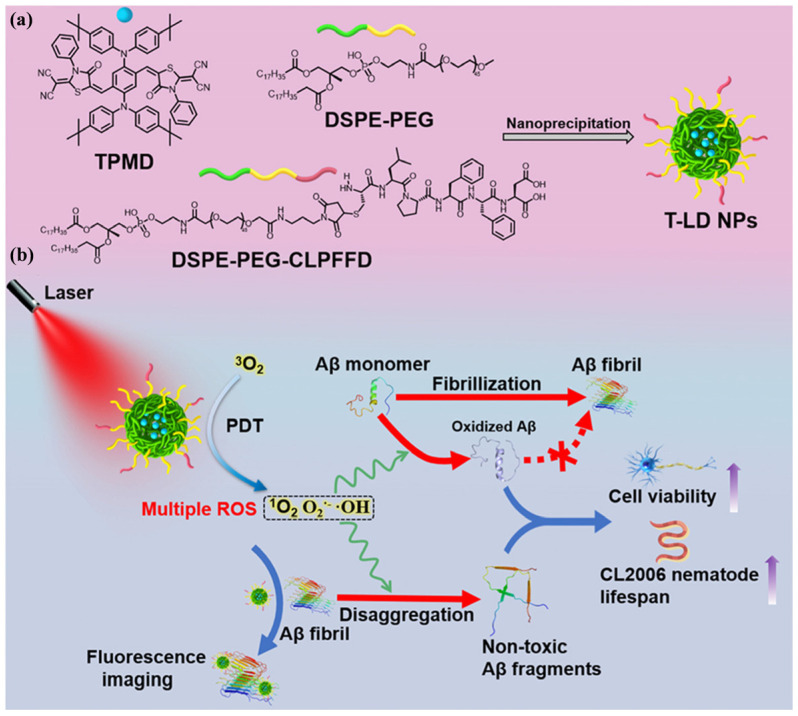
(**a**) Chemical structures and synthesis of T-LD NPs. (**b**) Schematic illustration of the fluorescence imaging of Aβ and the generation of ROS under laser irradiation for inhibiting Aβ aggregation and disaggregating Aβ fibrils. Reprinted with permission from ref. [171]. Copyright 2023 Royal Society of Chemistry.

Through feedback from in vivo monitoring results, the directional and precise development of drugs can be guided by highly sensitive imaging of tracking technology. Wang et al. designed an AIE-based nanotheranostic for precise AD therapy (Figure 28) [170]. The nanotheranostic showed brain-targeting, fibril-degrading, and ROS-regulating properties. Two theranostic AIEgens with NIR-II emission (compound 3 and compound 6) were co-assembled with DSPE-TK-PEG to form nanodrugs or nanocomposites named Ang-AIE NCs. Compound 3 released from the nanodrugs could specifically inhibit the formation of Aβ fibrils and disassemble the Aβ plaques. The released compound 6 could scavenge the poisonous inflammation-associated ROS by Ce(III) and further alleviate the formation of Aβ fibrils. It is noted that the Ang-AIE NCs show the longest emission at 1350 nm, which can improve the sensitivity for determining the across-skull signal of Aβ plaques after crossing the BBB. The nanodrugs reversed the neurotoxicity and improved behavioral and cognitive behaviors in AD mice. In addition, Liu et al. reported AIE photo-oxidant nanoparticles (T-LD NPs) for imaging, inhibition, and disaggregation of Aβ (Figure 29) [171]. The T-LD NPs were prepared by the co-assembly of a hydrophobic AIE fluorogen TPMD and an amphiphilic polymer (DSPE-PEG)-modified Aβ-targeting peptide (LPPFD). Under laser irradiation, T-LD NPs catalyzed the generation of ROS to oxygenate Aβ, inhibiting Aβ fibrillization, disaggregating mature Aβ fibrils, and ultimately reducing Aβ-mediated neurotoxicity.

**Table 2:** AIE probes for diagnosis and treatment of AD.

AIE Probe	Ex. (nm)	Application	Ref.
Quinoline derivative	570	Imaging Aβ plaques in AD mouse brain	[152]
PD-BZ-OH/PD-NA-OH	486	3D mapping Aβ plaques in Tg mouse brain	[154]
PD-NA-TEG	365	Imaging Aβ plaques in mouse brain	[155]
Cou-AIE-TPP^+^	604	Imaging Aβ-induced neuronal cell mitochondria	[156]
AIE-CNPy-AD	455	Tracing Aβ deposits in AD model mice	[157]
TPE peptide	370	Monitoring of Aβ fibrillation	[162]
AIE glyconanoparticle	374	Detecting Aβ fibrils	[163]
G7-TBA	450	Monitoring Aβ aggregation and inhibiting its cytotoxicity	[164]
QM-FN-SO_3_	500	Mapping Aβ plaques in AD mouse brain	[165]
QM-FN-SO_3_	500	Mapping Aβ plaques in AD brain tissues and living mice	[166]
ROF2	442	Detecting Aβ aggregates of different sequences	[167]
DNTPH	488	Imaging/reducing Aβ plaques in AD mouse brain	[168]
Ang-AIE NCs	808	Imaging Aβ plaques in AD mouse brain and inhibiting its cytotoxicity	[170]
T-LD NPs	600	Imaging and scavenging Aβ plaques	[171]

Non-destructive NIR imaging-assisted phototherapy of AD can display the accurate location of Aβ aggregates, monitor the real-time accumulation of photoresponsive materials, and guide the phototherapy with enhanced therapeutic efficiency and reduced side effects. However, fluorescence (radiative relaxation) and photothermal or photodynamic effects (non-radiative relaxation) are two competing energy dissipation pathways, which makes it hard to achieve a well-balanced distribution. AIEgens with proper structures can be encapsulated in polymer shells or self-assembled into nanomaterials, simultaneously serving as promising imaging and PTT/PDT agents. Despite their unique properties and exciting applications, more AIEgens with multifunctionality and longer excitation wavelengths should be explored to enhance their tissue penetration depth and therapy efficiency. In addition, the hybrid nanocomposites of AIEgens with other PTT or PDT materials can endow therapeutic drugs with even more stunning performance and functionality.

## 5. Conclusions

In summary, we summarized the latest advances in PTT and PDT strategies for the diagnosis and treatment of AD through imaging and regulating Aβ aggregates. Different materials with unique properties have been used as PTT or PDT reagents to modulate Aβ aggregation. Although great achievements have been made, there are still some challenges that need to be addressed regarding the successful clinical translation of phototherapy. First, the limited tissue penetration depth of ultraviolet and visible light in vivo results in low phototherapy efficiency. It is crucial to explore more novel photoresponsive materials with high NIR-I or especially NIR-II absorption and fluorescence to achieve deeper penetration, larger maximum allowable exposure, and high imaging quality. Second, the biocompatibility and biodegradability of photoresponsive materials (especially inorganic materials), biosafety of the used light sources, and heat/ROS generated in the PTT/PDT process should be appropriately considered and evaluated. Different photoresponsive materials exhibit distinguishing side effects, toxicity, biological distribution, and pharmacokinetic behavior. In this aspect, covalent organic frameworks may be promising candidates for AD diagnosis and therapy since they do not involve the use of metal ions, and the ROS-induced cell damage and tissue toxicity could be reduced. Third, it is worth noting that the low BBB permeability of some photoresponsive materials may make it difficult to reach the brain, severely limiting their clinical treatment efficacy. To overcome this issue, cell penetrating peptides can be used to modify materials and increase BBB permeability. In addition, focused ultrasound, magnetic fields, and electric fields can also be used to enhance BBB permeability of drugs, effectively assisting targeted delivery in the brain. Fourth, the majority of phototherapy in AD treatment is single modality (PTT or PDT), which rarely provides satisfactory therapeutic results because of its intrinsic shortcomings. For this view, novel modulators could be designed to develop imaging-guided PTT-PDT dual-modality phototherapies. Moreover, AIEgens and their hybrids with multifunctionality, such as target recognition, fluorescence imaging, and phototherapy, should be innovatively designed. Finally, other pathological markers can be regulated by PTT and PDT strategies, such as hyperphosphorylated tau protein, elevated levels of copper ions and ROS, and neuroinflammation. It is expected to design multiple therapeutic strategies that combine two or more functions in one platform, such as PTT, PDT, drug release, immunotherapy, etc. We hope that this review can provide a reasonable overview of the current development of phototherapy methods and lighten their clinical translation for AD.

## Data Availability

Not applicable.
